# Abnormal task modulation of oscillatory neural activity in schizophrenia

**DOI:** 10.3389/fpsyg.2013.00540

**Published:** 2013-08-27

**Authors:** Elisa C. Dias, Stephan Bickel, Michael L. Epstein, Pejman Sehatpour, Daniel C. Javitt

**Affiliations:** ^1^Center for Schizophrenia Research, Nathan Kline Institute for Psychiatric ResearchOrangeburg, NY, USA; ^2^Department of Neurology, Albert Einstein College of MedicineBronx, NY, USA; ^3^Division of Experimental Therapeutics, Department of Psychiatry, Columbia College of Physicians and SurgeonsNew York, NY, USA

**Keywords:** schizophrenia, oscillations, alpha, beta, AX-CPT, cognitive, motor preparation

## Abstract

Schizophrenia patients have deficits in cognitive function that are a core feature of the disorder. AX-CPT is commonly used to study cognition in schizophrenia, and patients have characteristic pattern of behavioral and ERP response. In AX-CPT subjects respond when a flashed cue “A” is followed by a target “X,” ignoring other letter combinations. Patients show reduced hit rate to “go” trials, and increased false alarms to sequences that require inhibition of a prepotent response. EEG recordings show reduced sensory (P1/N1), as well as later cognitive components (N2, P3, CNV). Behavioral deficits correlate most strongly with sensory dysfunction. Oscillatory analyses provide critical information regarding sensory/cognitive processing over and above standard ERP analyses. Recent analyses of induced oscillatory activity in single trials during AX-CPT in healthy volunteers showed characteristic response patterns in theta, alpha, and beta frequencies tied to specific sensory and cognitive processes. Alpha and beta modulated during the trials and beta modulation over the frontal cortex correlated with reaction time. In this study, EEG data was obtained from 18 schizophrenia patients and 13 controls during AX-CPT performance, and single trial decomposition of the signal yielded power in the target wavelengths. Significant task-related event-related desynchronization (ERD) was observed in both alpha and beta frequency bands over parieto-occipital cortex related to sensory encoding of the cue. This modulation was reduced in patients for beta, but not for alpha. In addition, significant beta ERD was observed over motor cortex, related to motor preparation for the response, and was also reduced in patients. These findings demonstrate impaired dynamic modulation of beta frequency rhythms in schizophrenia, and suggest that failures of oscillatory activity may underlie impaired sensory information processing in schizophrenia that in turn contributes to cognitive deficits.

## Introduction

Schizophrenia is a severe brain disorder associated with widespread cognitive deficits that represent a core feature of the disorder. Although deficits have been studied most extensively with regard to higher order constructs such as attention, working memory, or executive processing, deficits are documented as well in basic sensory processing. For example, in the auditory system, patients show deficits in pitch processing and mismatch negativity which correlate with dysfunction in higher cognitive abilities (Javitt, 2009; Leitman et al., 2010; Kantrowitz et al., 2013). In the visual system, patients show impairment on non-linear gain mechanisms, leading to reduced contrast sensitivity particularly within the magnocellular visual system (Butler et al., 2001), as demonstrated using behavioral, neurophysiological, and fMRI-based approaches (Butler et al., 2005; Maris and Oostenveld, 2008; Martinez et al., 2012). These deficits in sensory processing suggest that patients with schizophrenia are impaired not only in how they process information, but how they experience the world (e.g., Leitman et al., 2007; Butler et al., 2009).

One of the most informative tasks for evaluation of visual sensory-level contributions to higher order cognitive impairment in schizophrenia is the AX-type continuous performance task (AX-CPT; Rosvold et al., 1956). In the AX-CPT, subjects attend to a sequence of individually presented letters and must respond whenever they view a letter “A” followed by a letter “X,” while ignoring all other sequences. In the most widely used version of the task (“AX-70” version) the majority of trials consist of AX sequences, so that the critical challenge of the task becomes to withhold response to all other sequences. Patients show reliable deficits both in detection of AX-sequences and rejection of sequences in which an invalid cue (letter other than “A,” collectively termed “B”), is followed by a target (“BX” trials), leading to a reduction in a signal-detection measure termed d'-context, which is calculated based upon relative percentages of errors for sequences AX (misses) and BX (false alarms; Cohen et al., 1999; Barch et al., 2001; Dias et al., 2003, 2011; MacDonald et al., 2003; Bickel et al., 2012).

In contrast to their increased error rates on BX trials, patients have relatively intact ability to withhold response to non-target stimuli even if they are preceded by a valid cue (“AY” sequences, in which “Y” represents letters other than “X”), suggesting that performance deficits in this task in schizophrenia primarily reflect impaired ability to utilize cue information to determine whether to press or not press in response to a correct target (“X”) stimulus. By contrast, when given a stimulus which by itself requires a non-response (i.e., “Y” target), patients show preserved response inhibition (Cohen et al., 1999; Barch et al., 2001; Dias et al., 2011), showing relatively intact levels of task engagement.

Initial theories of AX-CPT dysfunction in schizophrenia suggested that impairments were due to inability to maintain information during the cue-target interval, in keeping with dopaminergic theories of schizophrenia (Cohen and Servan-Schreiber, 1992). Subsequent studies, however, showed similar deficits both at both short and long ISI, suggesting that impairments resulted primarily from decoding of cue information itself, rather than maintenance over time (Lee and Park, 2005, 2006; Javitt et al., 2007). A similar pattern of deficit is observed in healthy volunteers during acute challenge with the N-methyl-D-aspartate receptor (NMDAR) antagonist ketamine, suggesting that performance deficits on this task may reflect underlying impairment of NMDAR-dependent circuits (Umbricht et al., 2000).

Early theories of AX-CPT dysfunction in schizophrenia also suggested that deficits reflected primarily impairments in ability to withhold responses when the global prepotency, based upon relative probability of AX and BX trials, was to respond. In contrast, we have more recently observed that patients show greatest deficits, compared to controls, in versions of the task when AX probability is low, so the global prepotency is to not respond to a target (“X”) stimulus (Dias et al., 2011). The availability of parametric versions of this task, in which the proportions of the different types of trial are systematically varied creating different expectancies of response, permits detailed assessment of mechanisms underlying performance deficits in schizophrenia, using both fMRI and event-related potential techniques.

In fMRI versions of the task, greatest attention has been paid to activation of frontal areas. In these studies, consistent deficits are observed in dorsolateral prefrontal cortex (DLPFC) and anterior cingulate cortex (ACC; Barch et al., 2003; MacDonald and Carter, 2003; Perlstein et al., 2003). However, a more recent meta-analysis shows that in controls there is also activation of posterior visual areas, such as the middle occipital gyrus, that is not observed in schizophrenia patients, suggesting sensory-level disturbance as well (Minzenberg et al., 2009).

The sensory involvement in AX-CPT performance deficits in schizophrenia have more recently been amplified by ERP studies focusing on relative generation of late potentials that localize to higher cortical regions such as anterior cingulate or DLPFC (e.g., N2, P3) as compared to early potentials that localize to sensory brain regions such as striate (V1) and extrastriate (e.g., V3a) visual regions (e.g., P1, N1). The greater temporal resolution of ERP vs. fMRI, moreover, permits greater isolation of responses to cue- vs. target-stimuli.

**Figure 1:**
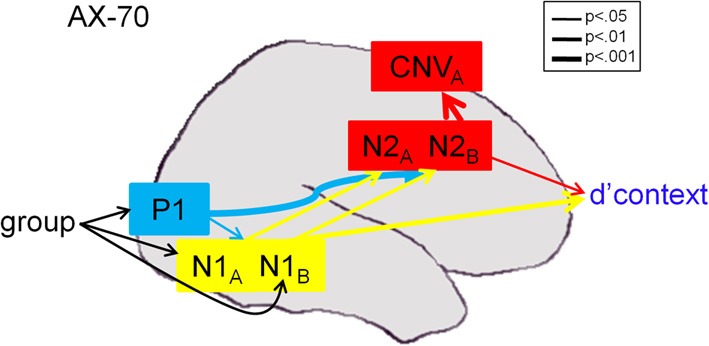
**Path analysis results in the AX-70 task variant [from Dias et al. (2011), online only materials]**. Component variables were overlaid on a schematic brain based upon generator locations derived from source analysis (Dias et al., 2003), monkey intracranial recordings (Dias et al., 2006), and prior fMRI studies (Barch et al., 2003). Arrows reflect significant statistical associations as shown by path analysis, with thickness of arrow representing strength of connection. CMIN/DF of the model was 1.109, and RMSEA was 0.052. For statistics, P1 values were collapsed across A- and B-cues, which were not significantly different (*p* > 0.2). For more information, see Dias et al. (2011).

As expected, significant deficits were observed even in response to cue stimuli, supporting concepts that dysfunction was related to impaired cue utilization (Dias et al., 2011). Moreover, deficits were observed in generation of both late ERP components, such as N2, P3, or contingent negative variation (CNV), that localize to frontal brain regions (e.g., ACC, DLPFC) as well as early ERP components such as P1 and N1 that localize to primary and secondary visual regions (Dias et al., 2011). In connectivity analyses, impaired performance, as reflected in d'-context, was associated with activity both within sensory and frontal brain regions (Figure 1). However, frontal deficits were, in turn, driven in part by failures in sensory processing, primarily within the magnocellular visual system, with little additional illness-related impairment in frontal function.

In the present study, AX-CPT deficits in schizophrenia are analyzed using a “time-frequency” approach to EEG data analysis, which utilizes trial-by-trial information to analyze the functional basis of impaired ERP generation. In the time domain approach, responses to individual stimuli or events are analyzed as a function of time, and are measured relative to a pre-stimulus baseline, which is presumed to reflect absence of activity.

By contrast, frequency domain approaches are based upon the concept that the brain is constantly engaged in ongoing, rhythmic activity, and that individual sensory events or cognitive demands not only induce new brain activity but also modulate ongoing brain activity. The amplitude of scalp-recordable rhythmic activity is dependent upon both the amplitude of activity within local oscillatory ensembles in the brain, and the degree to which ensembles are synchronized in time, with greater synchrony leading, in general, to greater scalp-detectable activity.

In order to quantify event-related effects on oscillatory processes, two separate measures are extracted in frequency domain approaches: first, the response power across EEG frequency bands for each individual event, termed “induced” power and second, the consistency of response across stimuli, termed “intertrial coherence” (ITC) or phase locking. The combination of these two measures provides a measure of “evoked” power, similar to responses measured in more common time-domain approaches. Increases in induced power may reflect either stimulus-driven activation, or event-related synchronization (ERS) of ongoing activity. In contrast, reductions of single-trial power are presumed to reflect primarily event-related desynchronization (ERD) of ongoing neural activity. Analysis of these single-trial events thus provides information that is not detectable using more traditional time domain approaches.

In the frequency domain approach, analyses are typically performed using pre-defined frequency bands which are characterized based upon order of discovery. Ongoing research is attempting to determine functional roles of the different rhythms and their interactions. Thus, the alpha frequency band (8–14 Hz) was first described in the 1930's and has been more recently shown to be related with mechanisms of alertness (Klimesch et al., 1998), attention (Foxe et al., 1998; Worden et al., 2000; Thut et al., 2006; Lakatos et al., 2008; Gould et al., 2011; Rohenkohl and Nobre, 2011), and inhibition (Klimesch et al., 2007).

The beta frequency band, (~14–30 Hz) has been associated with sensory-motor activity (Pfurtscheller, 1981; Schoffelen et al., 2008), sensory gating (Hong et al., 2008), and attention (Gross et al., 2004). Oscillations in the high frequency gamma band have been proposed as mechanisms for binding different visual features of objects (Gray et al., 1989), attention (Fries et al., 2001), and memory (Tallon-Baudry et al., 1998), and in the lower frequency theta band have been linked to hippocampal functions such as learning and memory (Buzsaki, 2002) and also entrainment of higher frequencies and sensory selection (Lakatos et al., 2008; Schroeder and Lakatos, 2009).

Consistent with this hypothesized role of alpha/beta frequency activity, we have previously demonstrated in a group of healthy individuals that cue stimuli in the AX-CPT elicit task-related reductions in alpha/beta activation during the cue and evaluation period, which are hypothesized to reflect the bringing of these regions “on-line” to permit them to process cue-related information. Similarly, we observed beta reductions over motor regions in response to valid cues, particularly in task versions that were most likely require a motor response (Bickel et al., 2012). The present study analyzes these events in patients with schizophrenia, in order to evaluate patterns of oscillatory analysis underlying impaired cue processing in the AX-CPT as a model system for evaluating mechanisms of overall visual system dysfunction in schizophrenia.

As in our prior analysis in healthy volunteers, separate analyses were performed within 3 discrete time windows: (1) a sensory response period (0–0.2 s) in which activity is driven by external stimuli and is localized to primary sensory regions, (2) a sensory evaluation period (0.2–0.5 s) in which sustained, continued event-related modulations of activity are observed primarily over sensory regions as well, and (3) a preparation interval (0.8–1.2 s) over which time activity appears modulated primarily by the likelihood of an upcoming target stimulus and requirement to respond. Based upon our prior time-domain analyses (Dias et al., 2011), we hypothesized that patients would show not only reduced stimulus-induced activity during the sensory response period, but also reduced stimulus-induced beta modulation during the sensory evaluation period. These reductions, which are not evident in time-averaged ERP data, provide additional mechanisms regarding neural basis of visual information processing impairments in schizophrenia.

## Methods

### Participants

Thirty patients meeting DSM-IV criteria for schizophrenia (SCZ), and 17 healthy controls (HCs) participated in the study. However, frequency analysis requires long epochs of EEG data, which results in the elimination of many trials, and so several subjects were rejected from the current analysis due to insufficient number of trials, and consequently the final number of subjects for this analysis is lower (18 SCZ, 13 HC). Two additional SCZ had to be removed from some analyses because of excessive line noise in one or more of the tasks.

The patients were diagnosed using the Structured Clinical Interview for DSM-IV and available clinical information, and were recruited from inpatient and outpatient facilities associated with the Nathan Kline Institute. HCs were recruited from the Nathan Kline Institute's Volunteer Recruitment Pool and were free from any SCID-defined Axis I psychiatric disorders. All participants had normal or corrected to normal visual acuity. All but one patient and one control were right-handed, as assessed with the Edinburgh Handedness Inventory (Oldfield, 1971). Participants were not included if they met criteria for substance or alcohol dependence in the last 6 months, or abuse within the last month. This study was approved by the Nathan Kline Institute internal review board. Additional subject characteristics, including neuropsychological scores, are presented in Table 1.

**Table 1:** **Subject characteristics**.

**Characteristic**	**Mean (*SE*)**		
	**Controls (*n* = *13*)**	**Patients (*n* = *17*)**	*P*-value^*^
Age, years	34.17 (2.2)	35.1 (2.0)	0.78
Sex (male/female)	10/3	17/0	
Chlorpromazine daily equivalent, mg		1232.9 (163.4)	
Personal SES	48.2 (2.8)	21.3 (2.1)	<0.001
Parental SES	50.4 (3.18)	41.1 (6.8)	0.16
BPRS total score		39.7 (2.0)	
SANS total score		33.8 (3.0)	
ILS standard score		35.6 (2.6)	
IQ (quick test)	112.8 (2.1)	96 (2.88)	<0.001
Duration of Illness		15.3 (1.8)	

Abbreviations *BPRS* *Brief Psychiatric Rating Scale (Overall and Gorham, 1962)* *ILS* *Independent Living Scales (Loeb, 1996)* *IQ* *intelligence quotient (Ammons and Ammons, 1962)* *SANS* *Scale for the Assessment of Negative Symptoms (Andreasen, 1984)* *SES* *socioeconomic status, measured by 4-factor Hollingshead Scale (Hollingshead, 1975)*.

* *P-values from t-tests*.

### Paradigm

The AX-CPT has been described in detail in previous papers (Dias et al., 2003, 2011; Bickel et al., 2012). In summary, a stream of cue-probe letter sequences were presented sequentially on a computer screen located 137 cm in front of the subjects' eyes using Presentation software (Neurobehavioral Systems, Inc., Albany, CA). Letters subtended ~2° of visual arc and were presented for 100 milliseconds, white on black, using Helvetica font.

The interval between onset of cue and onset of probe letters (SOA) was 1240 ms, and the interval between successive cue-probe sequences (ISI) was 1390 milliseconds. Subjects were instructed to respond by pressing a button with their right hand if the cue-probe sequence was the letter “A” followed by the letter “X,” while ignoring all other cue-probe sequences. Invalid cues, collectively referred to as “B” and invalid probes, collectively referred to as “Y,” were letters other than A and X. The response window following probe presentation was 1 s.

The task used in this study is different from some other versions of AX-CPT, in that a response is only required for the AX condition, whereas in other studies (Cohen et al., 1999; Barch et al., 2001; Braver et al., 2009), one response is required for correct and another for incorrect sequences. Thus, our task creates different expectations of a motor activity that are not present in other studies (Dias et al., 2003, 2011; Bickel et al., 2012).

Variations of the task were created by changing the proportions of each type of trial, generating different expectation of response. Three variants were used, and in each a specific cue-probe sequence was presented with 70% probability, while all other sequences were presented with 10% probability, in pseudorandom order (Table 2). Adjusting the prevalence of each cue-probe sequence allowed for selective manipulation of both the local and global prepotency to respond. Thus, the variant in which 70% percent of the trials were sequence A-X (named AX-70) created a global prepotency to respond to the probe X, and the subject had to use the information from the cue “B” to inhibit incorrect responses to the X in B-X sequences. In the variant with 70% of A-Y trials (AY-70), the subjects did not prepare a response following cue A, as most probes were invalid, and thus had to rapidly prepare a response when the target probe X was shown. And in the third variant, BX-70, the subject has mostly invalid trials, but once a cue A was shown, they had a 50% chance of having a response. The probabilities generated by the different task variants are detailed in Table 2.

**Table 2:** **Trial type probabilities for each AX-CPT task variant**.

**Task**	**Trial type**				**Global prepotency**	**P(A)%**	**P(X)%**	**P(X|A)%**	**Local prepotency**	**Probability ratio**
	**AX**	**BX**	**AY**	**BY**						
	**Go**	**No-Go**							**(After cue A)**	**(P(X|A)/P(A))**
AX-70	70	10	10	10	Go	80	80	87.5	Go	1.09
AY-70	10	10	70	10	No-Go	80	20	12.5	No-Go	0.16
BX-70	10	70	10	10	No-Go	20	80	50	Go = No-Go	2.50

Abbreviations *P(A)* *probability of cue A* *P(X)* *probability of target X* *P(X|A)* *probability of target X, given cue A*.

Each subject performed 6 blocks of 93 trials for each of the 3 tasks, totaling 1674 trials. In most cases, task AX-70 was presented first because this was the condition of primary interest. Task order did not significantly affect performance either across or within group [main effect of order: *F*_(3, 41)_ = 0.25, *P* = 0.86; group vs. order interaction: *F*_(3, 41)_ = 0.76, *P* = 0.52]. Subjects took many breaks between blocks and did not report abnormal fatigue during the tasks.

Performance was indexed by a modification of the signal detection theory measure of sensitivity d', called d'context, that calculates the separation between the means of the signal and noise distributions, and is calculated from measurements of the hit rate and false-alarm rate: d' context = *Z*(hit rate in AX trials) − *Z*(false alarm rate in BX trials), where *Z* is the inverse of the cumulative Gaussian distribution (Barch et al., 2001). A higher d' indicates that the signal can be more readily detected, and by restricting the analysis to AX and BX trials, the effect of cue encoding on the task performance can be indexed.

### EEG

**Figure 2:**
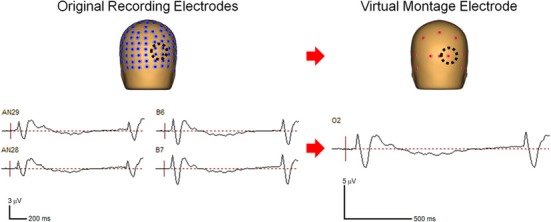
**Comparison of the grouped data at four original recording electrodes (left panel) and the virtual electrode representing that scalp region (right panel)**.

Continuous EEG data were originally collected and digitized at 512 Hz with an ActiveTwo system (Biosemi, Amsterdam, the Netherlands) using a 168 channel high-density cap as this montage afforded us the excellent spatial resolution required for source localization of the effects. However, for the purpose of the frequency analysis of scalp data, such a high electrode density is less critical. We therefore reduced the redundancy of the scalp electrode signatures by interpolating the data to a 27-channel virtual montage, referenced to the center of the head, for data analysis using BESA (Brain Electric Source Analysis, MEGIS Software GmbH, www.besa.de, Gräfelfing, Germany; Figure 2). By doing so we reduced the influence of a reference electrode. We further combined the data from a subset of virtual electrodes to create larger regions of interest, specified below, to investigate activity over scalp regions of interest based on a priori hypotheses, guided by previous investigations (Dias et al., 2011; Bickel et al., 2012).

Continuous data was epoched from −300 to 1550 milliseconds surrounding the digital tags that marked the onset of the stimuli, and band pass filtered (0.05 to 100 Hz). Trials with activity exceeding ± 120 microvolts, with eye blinks, or with incorrect responses, were eliminated. Occasional noisy channels were substituted by interpolated data from neighboring channels. Subjects with fewer than 40 correct trials were removed from the analysis.

In most cases, epochs were baseline-corrected to the average of the pre-stimulus period −300 to −100 before stimulus onset, but the data were also analyzed without this correction to examine absolute power. The data were re-referenced to the average reference.

The signal in each individual trial was decomposed into the time-frequency domain using the complex demodulation procedure implemented in BESA for a frequency window of 4–50 Hz. Frequencies were sampled in 2-Hz steps, and time in 25 millisecond steps.

Further analyzes were performed using MATLAB (Mathworks, Natick, MA), with Fieldtrip (http://www.ru.nl/fcdonders/fieldtrip/) and custom written scripts. The data for single trials was grouped by subject, to allow statistical analyses of the effects, and then, for the figures, into a grand average of all subjects in each group.

**Figure 3:**
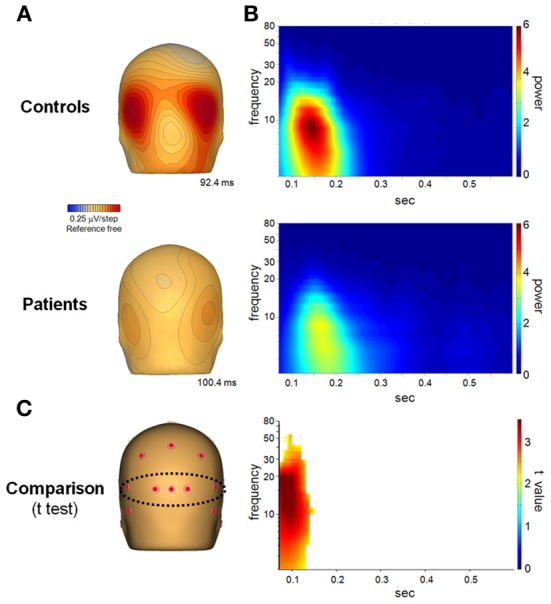
**(A)** Spatial distribution of the P1 component of the event related potential on the scalp, seen from behind the head, on both healthy controls (top panel) and schizophrenia patients (bottom panel). **(B)** Power of the evoked potential, obtained by extracting the frequency components of the averaged trials. **(C)** *T*-test comparison between patients and controls. The results are masked to only show *t*-values with a *p* < 0.05.

**Figure 4:**
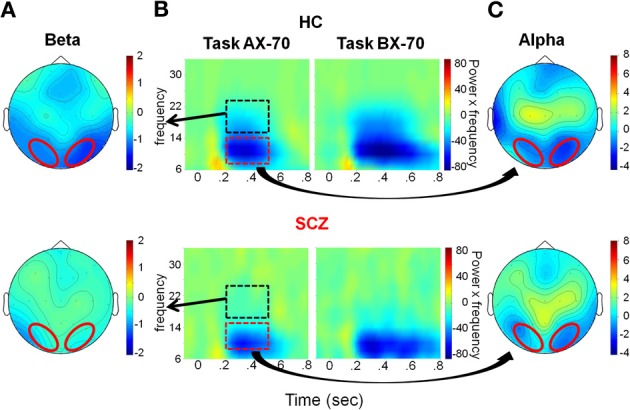
**Evaluation window. (A)** Top view of the spatial distribution of beta induced power during the evaluation period, for task AX-70; the red oval represents the scalp region in the occipito-parietal region that was used for the statistical analysis. The data for healthy controls are on top panels and for schizophrenia patients on the bottom panels. **(B)** Time-frequency distribution of activity in the occipito-parietal region following cue A onset (time 0), adjusted for power of the different frequencies (i.e., power multiplied by the specific frequency), for tasks AX-70 (left) and BX-70 (right). The black dotted rectangle represents the time-frequency window used for the analysis of beta power, and the red dotted rectangle represents the window for the alpha power. **(C)** Top view of the spatial distribution of the alpha induced activity for task AX-70.

A separate analysis was done, for comparison, by extracting the frequency information from the previously grouped data. This provided the power of the evoked potentials, shown in Figure 3 for task AX-70, cue A, for both SCZ and HC, as opposed to the induced activity that is observed when analyzing the power of the frequencies in single trials (Figures 4, 7). For the spatial representation of the evoked potentials shown in Figure 3A, the data were analyzed in the time domain with a low pass 6th order Butterworth filter at 35 Hz and baseline correction from −100 to 0 ms before stimulus onset.

For illustration purposes only, to better display the effects on the various frequency bands in the same time-frequency plots (Figures 4, 7), and counter the effect of the power reductions in the higher frequencies, the power in each 2 Hz frequency band was multiplied by the frequency it represented. Data analysis was performed on non-multiplied data.

To localize the sources of the activity recorded on the scalp, multi source beamformer (MSBF) was performed using the BESA source coherence module, by calculating the cross spectral density matrix of the specific frequency and time ranges of interest, for the grand mean average of each condition for each group, and computing the output power of the beamformer at each grid location. The figures (Figures 6, 9) show the location of the respective grid maximums plotted on the BESA standard anatomical MRI.

### Statistical analysis

Frequency boundaries used in this analysis were similar to those used in our previous manuscript (Bickel et al., 2012) and other published literature (Pfurtscheller, 1981; Farmer, 1998; Worden et al., 2000; Kelly et al., 2006; Uhlhaas and Singer, 2010), while avoiding overlap of the frequency ranges. Thus, we analyzed activity in the frequencies bands: theta (4–6 Hz), alpha (8–14 Hz), beta (16–24 Hz), and gamma (30–50 Hz). We did not analyze the data above 50 Hz.

We focused our analysis on three time windows: sensory, evaluation, and preparation (Bickel et al., 2012). The sensory window (0–0.2 s) was meant to capture the initial sensory volley of activity, including sensory ERPs P1 and N1; the evaluation window (0.2–0.5 s) was meant to capture the encoding activity; and the preparation window (1–1.3 s) captured the activity in preparation for the appearance of the probe.

**Figure 5:**
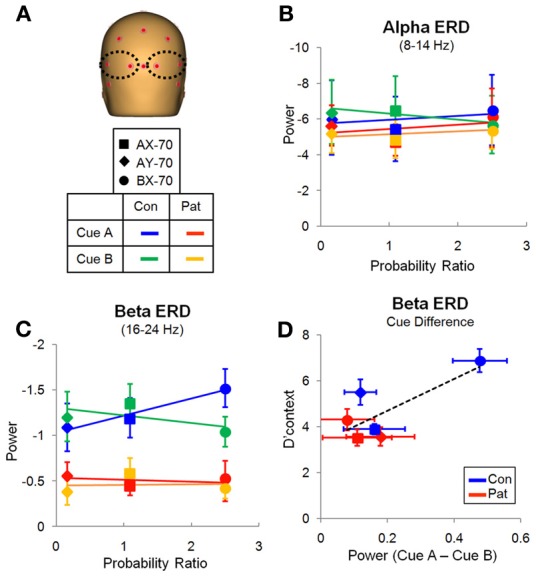
**Evaluation window. (A)** Location of the electrodes over the occipito-posterior scalp from which the data was derived. **(B)** Variation of the alpha ERD with the probability ratio for all three task variations, for cue A. There were no significant effects. **(C)** Variation of the beta ERD with probability ratio for all three task variations, for cue A (Table 2). There was a significant group difference and an interaction of task by cue. **(D)** Relation of the difference in beta ERD between cue A and B and performance measured by d' context, with linear trendline for all data points.

Regions of interest for analysis were chosen with basis on previous published data. For analysis of the activity of alpha and beta frequency bands over occipito-parietal cortex, data was obtained from an occipital parietal region, including virtual electrodes O1, O2, P7, and P8, of the 27 electrode montage (Figure 5A). Preparation beta was obtained from a left antero-medial region, including virtual electrodes FZ and F3, based on previous findings (Pfurtscheller, 1981; Alegre et al., 2006; Ritter et al., 2009; Bickel et al., 2012) (Figures 7, 8D). In addition, also based on results from our exploratory analysis on that study (Bickel et al., 2012), we analyzed the activity in the beta frequency range in the occipito-parietal region. Theta and gamma activities were examined both from an antero-medial region, including virtual electrodes FZ, F1, and F3 and from the occipito-parietal region.

Repeated measures ANOVA, with subsequent paired *t*-tests were used to assess significance. The model applied to each frequency band included task (AX-70, AY-70, or BX-70), cue type (A or B) and period (sensory, evaluation, preparation) as within-group factors and group (patient, control) as between-group factor. A secondary analysis was done for each period. All tests had a preset level of significance of *p* < 0.05. The commercial program SPSS (version 15) was used to compute the statistical tests. For the *t*-test analysis of the evoked time-frequency data, a cluster-based test was used as implemented in the Fieldtrip toolbox (Maris and Oostenveld, 2007). This approach controls for multiple comparison testing when computing statistics across multiple channels, and/or frequency and time points, and is further described in Bickel et al. (2012). A cluster level alpha of 0.05 was used.

## Results

### Behavioral results

AX-CPT performance by these subjects has been reported previously as part of a larger study (Dias et al., 2011). In this subgroup, behavioral findings remained unchanged from the larger group, including reduced performance accuracy across all trial types [*F*_(1, 27)_ = 12.53, *p* = 0.001] and prolonged reaction times [*F*_(1, 27)_ = 27.65, *p* < 0.0001] across tasks (Table 3). As in the larger sample, patients showed greatest deficits in the BX-70 version of the task lowest levels of deficit in the AX-70 version, leading to a significant group × task interaction [*F*_(2, 26)_ = 6.61, *p* = 0.005]. As in the larger group, performance deficits across task versions were due both to decreased hits on valid cue, correct target (AX) trials and false alarms to invalidly cued, correct targets (BX) trials.

**Table 3:** **Behavioral results**.

	**Reaction time (SEM) (ms)**		**D'context (SEM)**	
	**Controls**	**Patients**	**Controls**	**Controls**
AX-70	262 (22)	375 (20)	3.9 (0.3)	3.5 (0.4)
AY-70	372 (22)	508 (20)	5.5 (0.6)	3.6 (0.4)
BX-70	290 (20)	428 (18)	6.9 (0.5)	4.3 (0.5)

### EEG results

ERP responses were analyzed only from trials associated with a correct behavioral response. As previously (Bickel et al., 2012), separate analyses focused on activities within sensory (0–0.2 s), evaluative (0.2–0.5 s) and response preparation (0.5–1.0 s) time windows.

#### Sensory window (0–0.2 s)

In the sensory window, the primary averaged activity consisted of a bi-occipital positivity centered at 100 ms (Figure 3A). Evoked power of this activity was primarily in the theta frequency range, though it extended into higher frequencies for both groups (Figure 3B). Activity in patients was significantly reduced, as shown by *t*-tests between groups, followed by a cluster analysis (Figure 3C). The main deficits in patients vs. controls were observed in the initial stage of the response, consistent with the selective reduction of fast (e.g., magnocellular) input to the cortex.

Single-trial analysis showed additional power in the beta frequency range (16–24 Hz), with a significant group effect [*F*_(1, 27)_ = 14.6, *p* = 0.001], and a task by cue interaction [*F*_(2, 26)_ = 7.369, *p* = 0.003]. There were no interactions with group, suggesting that although power was reduced in patients, there was a similar modulation of beta activity between groups in this period. For induced power of oscillations in the alpha and gamma frequency bands there were no effects of task, cue or group, and no significant interactions in this period.

#### Evaluation window (0.2–0.5s)

In the evaluation time window, the main task-related modulations of oscillatory activity consisted of reductions in both alpha (8–14 Hz) and beta (16–24 Hz) frequency ranges, consistent with ERD of ongoing alpha/beta activity. The ERD started shortly after offset of the sensory ERP and persisted for variable duration depending upon task condition, with shortest duration in the AX-70 and AY-70 conditions, and longest duration in the BX-70 condition (Figure 4B). ERDs in both the alpha and beta frequency ranges were strongly localized over parieto-occipital scalp, suggesting modulation of activity within primary and secondary visual areas (Figures 4A,C).

Despite similar timing of the posterior alpha and beta activity, differential deficits were observed in patients vs. controls for the different frequency bands. In the alpha frequency range, patients and controls showed reductions in single-trial power that were large and that varied little across task versions (Figure 5B). On ANOVA, there were no significant main effects of group [*F*_(1, 27)_ = 0.146, *p* = 0.706], cue-type [*F*_(1, 27)_ = 0.011, *p* = 0.917], task [*F*_(2, 26)_ = 0.220, *p* = 0.804] or group X task interaction [*F*_(2, 26)_ = 0.186, *p* = 0.832].

By contrast to lack of cue/task effects or between-group difference in degree of alpha reduction across groups, there were highly significant effects of these parameters on beta ERD during the same evaluation latency range. Patients showed an overall reduction in magnitude of the beta ERD, resulting in a highly significant main effect of group [*F*_(1, 27)_ = 10.160, *p* = 0.004]. In controls, the magnitude of the beta ERD to A cues was smallest in the AY-70 task condition, intermediate in the AX-70 condition and greatest in the BX-70 condition (Figure 5C).

An opposite pattern of effects was observed for B cues, leading to a significant task version by cue type interaction [*F*_(2, 26)_ = 4.801, *p* = 0.017], although there were no significant main effects of task [*F*_(2, 26)_ = 0.603, *p* = 0.555] or cue [*F*_(1, 27)_ = 1.194, *p* = 0.284]. Patients showed a loss of this modulation by task, which resulted in a trend toward a 3-way task X group X cue interaction [*F*_(2, 26)_ = 2.569, *p* = 0.096]. Follow up ANOVAS for each group confirmed this finding, with controls showing significantly modulated beta ERD by task and cue [*F*_(2, 11)_ = 10.790, *p* = 0.003], reflecting differential information values of the cues in the different tasks, whereas patients did not [*F*_(2, 14)_ = 1.204, *p* = 0.329].

The pattern of activity in controls mirrored the probability ratio of the response, i.e., the likelihood of having to make a response (sequence AX), given the task (global prepotency) and the cue (local prepotency; Table 2), with beta ERD to “A” cues increasing with greater probability of requirement for an upcoming response (Figure 5C).

In addition, the BX-70 task, in which controls had the best performance, as indexed by d'-context, also showed a larger difference in beta ERD between cues (CueA – CueB), and this difference was reduced in patients, as was d'-context (Figure 5D). This suggests that the beta ERD may carry information differentiating the cues that contributes to task performance.

Given the orientation of visual generators, a reflection of the occipital activity was observed as well over anterior scalp regions (Figures 4A,C). The phase of the beta activity in this area was tested and, as expected, this activity was in counter-phase to the occipital activity, suggesting that the measured activity represented opposite poles of the same dipole. Moreover, amplitude of the frontocentral activity varied in parallel to the occipital activity across cue and task conditions, as assessed by Pearson correlations between the beta ERD power in anterior and posterior scalp regions throughout the trial (0–1.2 s). For example, for task AX-70, correlations between anterior and posterior beta ERD were all highly significant (*p* < 0.0001) both for patients and controls (controls, cue A, *r* = 0.656; cue B, *r* = 0.803; patients, cue A, *r* = 0.844, cue B, *r* = 0.822).

**Figure 6:**
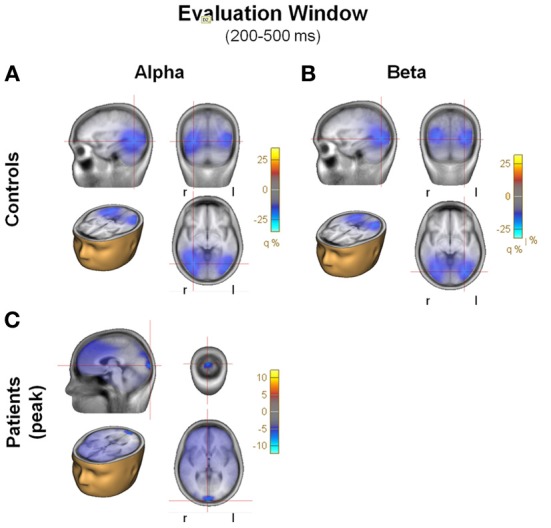
**(A)** Beamformer images of the sources of activity for alpha and beta during the evaluation window, plotted on the BESA standard anatomical MRI. Each panel includes 4 images of the peak of the activity: saggital, coronal, horizontal in head, horizontal. Blobs represent location of the grid maximums. Note the large differences in scale between controls and patients. **(A)** Alpha, controls; **(B)** Beta, controls; **(C)** Alpha, patients.

Consistent with this configuration of surface activity, in controls source analysis localized the main sources for the alpha and beta ERDs during the evaluation period in posterior regions of the brain, in the visual areas, with an emphasis on inferotemporal regions (Figures 6A,B). For patients, there was no detectable source at the same location (not shown), though for alpha ERD only there was a weaker source more localized to the occipital pole (Figure 6C).

Despite the smaller magnitude of the beta ERD, patients showed similar pre-stimulus absolute amplitude to controls within both the alpha (Pat: 17.708 ± 1.744; Con: 14.386 ± 1.963; *p* = 0.217) and beta (Pat: 3.333 ± 0.446; Con: 3.843 ± 0.417; *p* = 0.440) frequency ranges. No significant cue-induced modulation was observed within either theta or gamma frequency ranges during the evaluation period in any task version for either group. Furthermore, no difference in absolute amplitudes of theta or gamma activity was observed across groups.

#### Preparation window (0.8–1.2 s)

**Figure 7:**
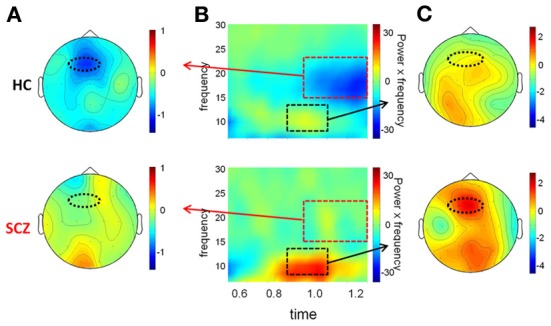
**Preparation window. (A)** Top view of the spatial distribution of beta induced power in the preparation period for task AX-70; the black oval represents the scalp region in the left fronto-central region. In **(A)**, **(B)**, and **(C)**, the data for healthy controls are on top panels and for schizophrenia patients on the bottom panels. **(B)** Time-frequency distribution of activity in the left fronto-central region adjusted for power of the different frequencies, for task AX-70. The red dotted square represents the time-frequency window used for the analysis of beta power, and the black dotted square represents the window for the alpha power, a little earlier (0.9–1.1 ms after cue onset). **(C)** Top view of the spatial distribution of the alpha activity for task AX-70.

In the preparation window, a significant reduction in beta activity was also observed in the control group. However, as opposed to the beta ERD observed in the evaluation window, beta ERD during the preparation window was maximal over frontocentral scalp, with asymmetric distribution toward left motor/hand region. Amplitude and spectral width of the beta ERD increased progressively to the point of target presentation (Figure 7B).

**Figure 8:**
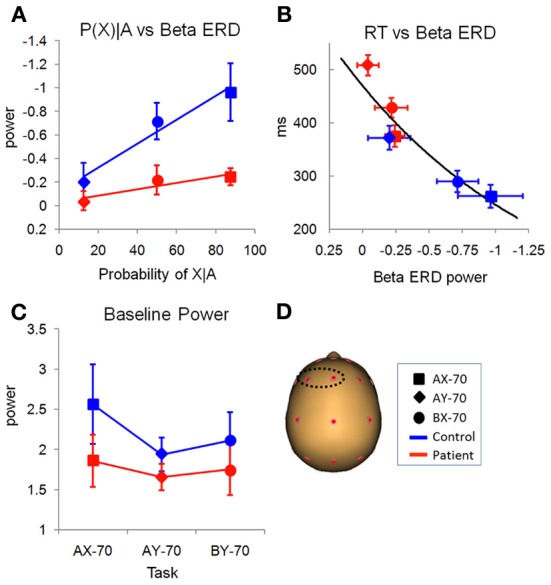
**Preparation window. (A)** Relationship of preparation beta ERD with the probability of target probe X, given cue A, for the different task variations (Table 2). There were main effects of task and group. **(B)** Relationship of preparation beta ERD power and reaction time (in milliseconds), showing that as beta ERD in the preparation window decreases, the reaction time increases. **(C)** Variation of ongoing baseline beta power with the different tasks that create different expectations of responses. In controls, task AX-70, that has a higher probability of response, has a larger power of beta than the other tasks. **(D)** Location on the scalp of the left antero-medial electrodes used for statistical analysis.

Furthermore, the pattern of the beta ERD across task versions was significantly different from the pattern of beta ERD in the evaluation window. Specifically, the magnitude of the beta ERD varied with the probability of a response following the presentation of cue A (Figure 8A). Thus, it was least in the AY-70 condition in which target (“X”) probability was low even after an “A” cue, intermediate in the BX-70 condition in which target probability was intermediate, and largest in the AX-70 condition, in which target probably was highest, leading to significant main effects of task [*F*_(2, 26)_ = 3.407, *p* = 0.049] and cue [*F*_(1, 27)_ = 14.22, *p* = 0.001] and of the cue by task interaction [*F*_(2, 26)_ = 6.354, *p* = 0.006]. This relationship was not present for cue B, which guaranteed that there was no need to prepare a response, independent of the upcoming probe type. Thus, the beta ERD in the preparation window appeared to track probability of response. In controls, the reaction time across tasks closely followed the pattern of the beta ERD, suggesting that the beta ERD represents response preparation (Figure 8B).

**Figure 9:**
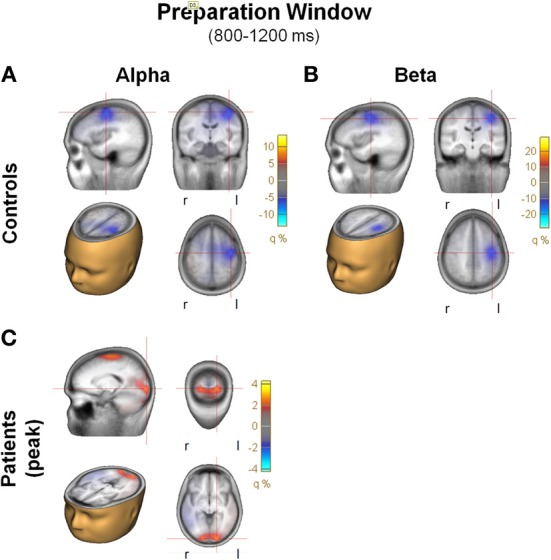
**(A)** Beamformer images of the sources of activity for alpha and beta ERD during the preparation window, plotted on the BESA standard anatomical MRI. Each panel includes 4 images: saggital, coronal, horizontal in head, horizontal. Blobs represent location of the grid maximums. Note the large differences in the scales, especially between controls and patients. **(A)** Alpha, controls; **(B)** Beta, controls; **(C)** Alpha, patients.

In patients, the magnitude of the beta ERD was dramatically reduced leading to a highly significant main effect of group [*F*_(1, 27)_ = 11.38, *p* = 0.002; Figure 7B, bottom panel]. Patients, like controls, showed increases in beta ERD magnitude across conditions as a function of target probability (Figure 8A). Although the differences were smaller overall than in controls, the group X task interaction was not significant [*F*_(1, 27)_ = 0.729, *p* = 0.492], suggesting a similar level of modulation. RTs for patients were significantly longer than controls across task versions [*F*_(1, 27)_ = 27.65, *p* < 0.0001], but were proportionate to the smaller beta ERDs (Figure 8B). Source analysis of the beta ERD in controls shows high localization to hand motor/premotor areas (Figure 9A). This is not observed in the model for the patients (not shown).

In addition, in the preparation interval (0.8–1.2 s) there was an increase in alpha power that was only marginally observed in controls but which was prominent in patients (Figure 7B) and preceded the beta ERD, so an exploratory analysis was performed. The activity showed a similar spatial distribution (Figure 7C), and, consistent with the visualized data, the increase in alpha power was significantly larger in patients than controls [*F*_(1, 27)_ = 4.81, *p* = 0.04]. A model of the sources of alpha activity in this period indicated that in controls the main source was also localized to the antero-medial region, in a similar in location to the beta activity, but was absent in this region in patients. The main peak of activity in patients had predominant occipital localization (Figure 9C).

As opposed to baseline activity over posterior scalp regions, baseline activity over left frontal scalp was modulated both as an effect of task and across group. Activity in this region showed a task effect [*F*_(2, 26)_ = 5.505, *p* = 0.010], and, although there was no main effect of group [*F*_(1, 27)_ = 1.155, *p* = 0.292], an interaction of task by group [*F*_(2, 26)_ = 3.619, *p* = 0.041] (Figure 8C). While for patients there was not much variation of the baseline, for controls there was an increase in beta baseline for task AX-70. Tests of within-subject contrasts showed that there were significant differences between the baselines for task AX-70 and AY-70 (*F* = 5.629, *p* = 0.025) and between AX-70 and BX-70 (*F* = 11.340, *p* = 0.002).

### Gamma

Analysis of baseline-corrected gamma activity over the occipito-parietal region showed no significant effects of group, cue or task, or any interactions of these factors in any of the periods. The same was true for the antero-medial region.

In the occipito-parietal region only, the baseline value of gamma showed an interaction of task by group [*F*_(2, 26)_ = 4.560, *p* = 0.020], which was driven by a modulation of absolute gamma power by task in controls [*F*_(2, 11)_ = 4.728, *p* = 0.033], but not in patients [*F*_(2, 14)_ = 2.424, *p* = 0.125]. There were no main effects of task [*F*_(2, 26)_ = 0.364, *p* = 0.698] or group [*F*_(1, 27)_ = 1.406, *p* = 0.246].

## Discussion

Cognitive deficits represent a core component of schizophrenia and a primary determinant of impaired long-term outcome. While such deficits are distributed throughout cortex, deficits are strongly manifest within early visual regions as well, and thus provide insights into mechanisms by which early visual deficits contribute to the overall cognitive dysfunction associated with schizophrenia. The present study utilizes evolving methods in time frequency analysis of ERP data to isolate neural patterns that may underlie the increasingly well-documented visual disturbances in schizophrenia.

Principal findings are two-fold: first, that significant additional activity can be detected in time-frequency analysis of single-trial ERP data than is apparent from analysis of average ERP data alone and, second, that deficits in stimulus evaluation and task performance in schizophrenia are primarily reflected in impaired modulation of beta activity across sensory, evaluation and motor preparation intervals. In contrast, relatively preserved activity was observed within other frequency bands, suggesting that deficits in beta generation may be a core component of schizophrenia and a tool for translational modeling of cortical dysfunction.

In visual tasks, such as the AX-CPT, primary evoked activity occurs within the theta frequency range. In intracortical recordings, this activity corresponds to a feedforward pattern of activation within primary (V1,V2) and secondary (e.g., V3a) regions, and thus most likely reflects feed-forward, thalamocortical activation mediated by magnocellular- and parvocellular-geniculocortical afferents. Stimuli used in this task are naturalistic (i.e., letters) and so do not isolate either magno- or parvocellular pathways. Nonetheless, the main difference between groups was early after cue presentation, supporting previous reports that the main deficit in patients is in the faster magnocellular input (Butler et al., 2005; Maris and Oostenveld, 2008; Martinez et al., 2012).

In addition to evoked activity, in cognitive tasks such as AX-CPT, stimuli lead to well-described modulations of ongoing activity that interact with task demands. These modulations occur with relatively large phase variability across trials, and so are not typically observed in traditional time-domain analyses. By contrast, in frequency domain analyses of single trial (induced) power, both event-related reductions in power are observed reflecting ERDs or increases in power reflecting primarily ERSs. Reductions in alpha/beta power, in particular, are thought to reflect local disinhibition of cortical regions, permitting them to engage in detailed stimulus processing. In addition, coherent beta activity across brain regions is thought to reflect interregional information transfer (Siegel et al., 2012).

In AX-CPT, we have previously observed significant beta desynchrony over visual areas during a stimulus evaluation period in healthy volunteers as well as beta desynchrony over motor regions during response preparation (Bickel et al., 2012). We have also observed that patients with schizophrenia are slower, and less accurate than controls in the AX-CPT task (Dias et al., 2011). The present study evaluated integrity of time-frequency modulations in patients with schizophrenia vs. healthy volunteers relative to their performance impairment. As expected, deficits were observed in both the evaluation and motor preparation latency ranges, reflecting disrupted neural activity, as described below.

### Evaluation window

In the evaluation latency range, controls showed a prominent, long-lasting ERD that involved both alpha and beta frequency ranges, and that persisted for variable time depending upon task. An unexpected finding in the patient group is that while they had marked reduction in magnitude of the beta ERD, the magnitude of the alpha ERD was unchanged.

In controls, alpha and beta modulations showed very similar source localizations, and thus likely represent operation of similar brain regions. Nevertheless, whereas activity in the beta range was significantly modulated as a function of cue and task, activity in the alpha range was not, suggesting that they may reflect operation of ensembles engaged in extraction of separate types of information. For example, alpha-blocking is observed even during processing of relatively primitive stimuli (e.g., flash, gabor), whereas in this study the stimuli are both complex and contain information related to their configural identity. Thus, the beta activity may be more related to processing of the complex stimuli such as letters, rather than simply as visual events.

Consistent with this hypothesis, in controls the magnitude of the beta ERD varied significantly across tasks, with inverse pattern for A and B cues. Moreover, the magnitude of the beta ERD was greatest when the information content of the stimulus was greatest relative to both global and local prepotencies. Thus, for task AX-70, cue A was more common than cue B and also indicated a higher probability of a response. In this case, the beta ERD was greater for cue B than cue A, consistent with the fact that cue B held more information in that it necessitated a decision to override the prepotent set to respond to a subsequent target stimulus.

By contrast, in task BX-70, cue B was more common and the global prepotency was to not respond even when a target was presented. In this case, an A cue would signal the need to override the global prepotency and thus was most informative with respect to successful task completion. The differential response within the alpha and beta frequency bands thus suggests that these reflect operations of spatially overlapping but functionally distinct neuronal ensembles, with alpha-frequency ensembles being primarily involved in stimulus detection, but beta frequency ensembles being involved in information extraction from the stimuli. The similar magnitude of the alpha modulation suggests that patients are appropriately detecting stimuli, but impaired in their ability to bring “on line” the specific ensembles needed to interpret the cues relative to task demands.

Other studies have also associated beta modulations with cognitive tasks (Tallon-Baudry, 2003; Pesonen et al., 2007; Haenschel et al., 2009), including beta activity in occipito parietal areas as in this study (Pesonen et al., 2007).

### Motor preparation

Beta activity in pre-central areas has been traditionally linked to motor control. Studies have shown that beta decreases prior to movement onset, and rebounds after termination, maintaining the activation during steady contraction (Pfurtscheller, 1981; Sanes and Donoghue, 1993; Crone et al., 1998; Farmer, 1998; Donner et al., 2009). It appears that during periods of low beta power the motor cortex is in a receiving state for new information or commands from other cortical areas (Crone et al., 1998; Bickel et al., 2012). Furthermore, since movements require only a small region of motor cortex (e.g., right index finger), the scalp-recorded reduction may represent a sharpening of the tuning curve for motor cortex in general, with suppression of those regions of motor cortex not necessary for the response but facilitation of regions particularly involved. Given the well-known association of beta modulation over motor cortex and motor response, it was possible to monitor task-related modulations of activity over antero-medial regions, and in particular left motor/premotor hand area relative to performance on the task.

In this study, as in our previous one with healthy subjects (Bickel et al., 2012), there was a gradual increase of beta ERD in the epoch preceding the potential target, proportional to the probability that a response might be required, that is, following A cues in the different tasks. This increase in beta ERD was also proportional to the reaction time, supporting the link between preparation beta and motor preparation (Bickel et al., 2012). Patients showed a much reduced beta ERD overall, though all trials examined resulted in a correct response. However, SCZ patients had significantly longer reaction times, suggesting that alternative, and possibly longer, neural pathway were recruited or, alternatively, weaker activity had accumulate before a response could be initiated. The model of the source of beta band activity in the preparation window showed no detectable activity in patients in the areas most activated in controls, i.e., the left antero-medial region, overlaying the motor/premotor cortex. In addition, patients had a stronger alpha ERS in the preparation window, that might represent activation of alternative pathways that contribute to task completion, but this remains to be tested more extensively. An increase in cortical synchronization in alpha frequencies (“high alpha,” 11–13 Hz), with a similar topography, has been previously shown in schizophrenia patients relative to controls, during the correct performance of a working memory task (De Vico Fallani et al., 2010). The authors also suggested this increase in synchronization could be related to compensatory mechanisms.

As in our previous study (Bickel et al., 2012), there was no significant gamma modulation during the task in either controls or patients, in the regions and for the range of frequencies we tested (30–50 Hz). The only gamma difference we observed was a variation of baseline activity with task in controls that was not present in the patient data. Recent studies have shown that gamma modulation in schizophrenia patients occurs mainly for higher frequency gamma (>50 Hz), for example in working memory (Haenschel et al., 2009) and visual encoding (Grutzner et al., 2013). Since our study was restricted to frequencies below 50 Hz, these reductions in high gamma would not have been detected in our study.

Although this study used relatively simple stimuli, and a relatively simple task, we would like to propose that similar deficits in beta activation may underlie other aspects of visual dysfunction. In addition, the fact that we observed deficits over motor cortex during the motor preparation period suggests that there is a generalized failure to modulate beta activity throughout task performance. The lack of coordination between brain regions, and lack of selective and sequential activation of appropriate brain regions may give rise to the cortical dysfunction that could underlie the profound impairment of schizophrenia patients.

The dissociation between beta modulations, which were severely impaired within both sensory and motor regions, and alpha and gamma modulations, which were relatively intact in schizophrenia, gives rise to a relatively specific pattern of neurophysiological impairment which can be used translationally as a relatively specific marker of cortical dysfunction in schizophrenia.

## Funding

This work was supported by the National Institute of Health (R37MH49334, P50MH086385 to Daniel C. Javitt).

### Conflict of interest statement

The authors declare that the research was conducted in the absence of any commercial or financial relationships that could be construed as a potential conflict of interest.
